# Quantitative PCR of ear discharge from Indigenous Australian children with acute otitis media with perforation supports a role for *Alloiococcus otitidis* as a secondary pathogen

**DOI:** 10.1186/1472-6815-12-11

**Published:** 2012-10-03

**Authors:** Robyn L Marsh, Michael J Binks, Jemima Beissbarth, Peter Christensen, Peter S Morris, Amanda J Leach, Heidi C Smith-Vaughan

**Affiliations:** 1 Child Health Division, Menzies School of Health Research, Darwin, Australia; 2 Institute of Advanced Studies, Charles Darwin University, Darwin, Australia; 3 Northern Territory Clinical School, Flinders University, Adelaide, Australia

**Keywords:** *Alloiococcus otitidis*, Otitis media, Acute otitis media with perforation, Indigenous Australian children, Bacterial load

## Abstract

**Background:**

Otitis media is endemic in remote Indigenous communities of Australia’s Northern Territory. *Alloiococcus otitidis* is an outer ear commensal and putative middle ear pathogen that has not previously been described in acute otitis media (AOM) in this population. The aims of this study were to determine the presence, antibiotic susceptibility and bacterial load of *A. otitidis* in nasopharyngeal and ear discharge swabs collected from Indigenous Australian children with AOM with perforation.

**Methods:**

Paired nasopharyngeal and ear discharge swabs from 27 children with AOM with perforation were tested by *A. otitidis* quantitative PCR (qPCR). Positive swabs were cultured for 21 days. Total and respiratory pathogen bacterial loads in *A. otitidis*-positive swabs were determined by qPCR.

**Results:**

*A. otitidis* was detected by qPCR in 11 ear discharge swabs from 10 of 27 (37%) children, but was not detected in paired nasopharyngeal swabs. *A. otitidis* was cultured from 5 of 11 qPCR-positive swabs from four children. All *A. otitidis* isolates had minimum inhibitory concentrations consistent with macrolide resistance. All *A. otitidis* qPCR-positive swabs were culture-positive for other bacteria. *A. otitidis* bacterial load ranged from 2.2 × 10^4^-1.1 × 10^8^ cells/swab (median 1.8 × 10^5^ cells/swab). The relative abundance of *A. otitidis* ranged from 0.01% to 34% of the total bacterial load (median 0.7%). In 6 of 11 qPCR-positive swabs the *A. otitidis* relative abundance was <1% and in 5 of 11 it was between 2% and 34%. The *A. otitidis* bacterial load and relative abundance measures were comparable to that of *Haemophilus influenzae*.

**Conclusions:**

*A. otitidis* can be a dominant species in the bacterial communities present in the ear discharge of Indigenous children with AOM with perforation. The absence of *A. otitidis* in nasopharyngeal swabs suggests the ear canal as the likely primary reservoir. The significance of *A. otitidis* at low relative abundance is unclear; however, at higher relative abundance it may be contributing to the associated inflammation. Further studies to better understand *A. otitidis* as a secondary otopathogen are warranted, particularly in populations at high-risk of progression to chronic suppurative otitis media and where macrolide therapies are being used.

## Background

Otitis media is endemic in remote Indigenous communities in Australia’s Northern Territory. In this population, middle ear infection begins in the first weeks and months of life [1] and often progresses to perforation and chronic suppurative otitis media (CSOM) [2]. Cross-sectional studies have reported perforation in up to 24% of Indigenous children in the Northern Territory [3], exceeding the rate of 4% recommended by the World Health Organization as requiring urgent public health attention [4]. Standard antibiotic therapies for acute otitis media (AOM) have limited effect in this population. A randomised controlled trial (RCT) of antibiotic treatments for AOM in Indigenous children reported clinical treatment failure in 50% and 54% of children treated with single dose azithromycin or 7 days amoxycillin, respectively (RD, -4%, [95% CI, -15% to 7%]; P = 0.504) [5]. High clinical failure rates following antibiotic treatment are likely due to the dense, polymicrobial nature of otitis media in this population [6].

*Alloiococcus otitidis* is a slow-growing, strictly aerobic, Gram-positive coccus [7]. Culture-based detection of this species requires prolonged incubation for 5-14 days in an aerobic environment [8-10], and thus, *A. otitidis* will not be detected by the standard culture conditions used to identify *Streptococcus pneumoniae* or *Haemophilus influenzae*.

*A. otitidis* has not previously been considered in AOM affecting Indigenous Australian children; although one Australian study reported 45% Indigenous children with otitis media with effusion (OME) had *A. otitidis*-culture positive middle ear specimens [8]. Qualitative PCR-based studies from other populations have reported *A. otitidis* in MEF collected by tympanocentesis from 20-61% of children with chronic OME [11-14] and 25-50% of children with AOM [13,15-17].

Despite its detection in middle ear fluid (MEF), a primary pathogenic role for *A. otitidis* remains controversial. Data supporting a pathogenic role for *A. otitidis* include its detection in MEF from children with OME [14,16,18] and AOM [17] (in the absence of recognised otopathogens), and its capacity to provoke an inflammatory response [18-21]. One study of children with AOM reported IL-8, IL-1-β and IL-6 levels in *A. otitidis*-positive MEF to be similar to that of *S. pneumoniae-*positive specimens [22].

The reservoir of *A. otitidis* associated with otitis media is unclear. *A. otitidis* has been detected by culture or PCR in 7-12% of nasopharyngeal swabs from paediatric and adult patients with upper respiratory infection or otitis media [9,16,23]. It has also been detected by culture or PCR in 14-83% of ear canal swabs from healthy volunteers [9,24-26], suggesting *A. otitidis* detection in MEF may reflect specimen contamination by normal canal flora. However, both PCR and culture-based studies have reported *A. otitidis* in MEF collected by tympanocentesis after careful cleaning and disinfection of the canal and tympanic membrane [8,14,22,27].

Although *A. otitidis* antibiotic minimum inhibitory concentration (MIC) breakpoints have not been defined [28], MIC levels consistent with macrolide resistance in *S. pneumoniae* have been reported for 11 of 20 (55%) [8] and 11 of 12 (92%) [29]*A. otitidis* isolates from two studies.

Azithromycin is an emerging AOM therapy for Indigenous Australian children [5]. Whether *A. otitidis* is associated with AOM in this population (and the significance of any association) is currently unclear. The aims of this study were: i) to determine the prevalence of *A. otitidis* in nasopharyngeal and ear discharge swabs from Indigenous Australian children with AOM with perforation; ii) to determine the antibiotic susceptibility of *A. otitidis* isolates to penicillin and macrolides; and iii) to determine the relative abundance of *A. otitidis* in polymicrobial specimens.

## Methods

### Ethical approval

Ethical approval for this study (HREC 07/85) was provided by the Human Research Ethics Committee of the Northern Territory Department of Health and Menzies School of Health Research, which includes an Aboriginal Sub-committee.

### Otitis media definitions

Otitis media definitions were as previously described [5]. AOM with perforation was defined as the presence of middle ear discharge for less than six weeks, and perforation covering less than 2% of the pars tensa of the tympanic membrane. Acute otitis media without perforation was defined as a bulging tympanic membrane and a type B tympanogram. OME was defined as an intact and non-bulging tympanic membrane and type B tympanogram.

### Specimens

This study retrospectively tested paired nasopharyngeal and ear discharge swabs collected at enrolment into a randomised controlled trial (RCT) of amoxycillin or azithromycin treatment for acute otitis media, conducted between March 2003 and July 2005 [5]. Eligible children were Indigenous, living in a remote community, between 6 months and 6 years of age, and had not received antibiotics in the 7 days preceding specimen collection [5]. Children were only selected for inclusion in the *A. otitidis* study if parental consent was given for their future use in related studies.

The RCT enrolled 320 children and included 49 children with a diagnosis of AOM with perforation. Of these 49 children, paired baseline nasopharyngeal and ear discharge swabs from 27 Indigenous children (median age 1.2 years, range 0.5-4.0 years) with AOM with perforation were available for *A. otitidis* testing. Four children had bilateral perforation. Thus, the sample set included 27 nasopharyngeal and 31 ear discharge swabs (58 swabs in total). As part of the original RCT, 12 children were treated with amoxycillin (50mg/kg/day in two divided doses for a minimum of 7 days) and 15 received azithromycin (30mg/kg as a single dose). Tympanic membrane perforation had not resolved in 21 of 27 children when reviewed 6-11 days after antibiotic treatment commenced. In the six remaining children, 3 had AOM without perforation (2 azithromycin treated) and 3 had OME (2 azithromycin treated) when reviewed.

Nasopharyngeal swabs were collected as previously described [5,30]. Ear discharge swabs were collected after the ear canal was cleaned to remove any visible debris and pus. The ear discharge was then sampled under direct vision (Welch Allyn Lumiview) by positioning the swab as close to the perforation as possible. Swabs were placed in 1mL skim milk-tryptone-glucose-glycerol broth [31] and frozen immediately at -20°C before being transported to the laboratory in a liquid nitrogen dry shipper [30] for long-term at storage -70°C.

### DNA extraction

Total DNA was extracted from the swabs as previously described [6] with some modification. Briefly, cellular material in a 200μL aliquot of each swab was pelleted by centrifugation. Pellets were resuspended with 200μL of enzymatic lysis buffer (containing 36.25mM phosphate buffer, 1mg/mL lysozyme (Sigma®), 0.75mg/mL mutanolysin (Sigma®), and 2mg/mL Proteinase K(QIAGEN)) then incubated at 56°C for 45 min. 10μL of 20% w/v sodium dodecyl sulfate (Amresco) was added, followed by gentle mixing at room temperature for 2 min. If the solution did not become clear, 20μL of 20mg/mL Proteinase K was added and the samples were incubated at 56°C for 10 min. 4μL of 100μg/mL RNase A (QIAGEN) was added and the samples were incubated at 70°C for 10 min. 200μL of 100% ethanol (Merck) was added and the samples were pulse vortexed for 15 s. DNA was then extracted using QIAamp DNA Mini Kit (QIAGEN) as per the manufacturer’s tissue protocol. DNA was eluted with 200μL of Buffer AE (QIAGEN).

For bacterial isolates, up to 100 colonies were harvested from purity plates and suspended in sterile water. Cells were pelleted by centrifugation and DNA was extracted as described above. The DNA concentration was determined using PicoGreen® reagent (Molecular Probes) as per the manufacturer’s instructions with fluorescence measured using a Victor3^TM^ 1420 Multi-label counter (Perkin Elmer) and WorkOut software (Perkin Elmer; version 2.5).

### General parameters for bacterial load qPCR assays

All qPCR assays were performed using Rotor-Gene 6000 real-time thermocyclers (Corbett Research). Quantitative standards were prepared by 1:10 serial dilution of reference isolate genomic DNA. qPCR raw data and standard curves were prepared using the Rotor-Gene 6000 software (Corbett Research; version 1.7). All standard curves were linear within the ranges described below. The qPCR efficiency and R^2^ values were calculated using the “Auto-find threshold” function with default settings. All standards and samples were tested in duplicate. Results were only accepted where duplicates differed by ≤0.5 cycles. Each assay’s limit of detection was defined as the lowest standard concentration at which specific amplification was detected in at least 95% of replicates [32].

### *A. otitidis* qPCR

The *A. otitidis* qPCR used primers previously described by Hendolin *et al.*[33] (Table 1) which amplify a 265-bp *A. otitidis*-specific region of the 16S rRNA gene [GenBank: NR_026088] between positions 437-702, based on *E. coli* numbering. The qPCR was done using a SensiMix^TM^*NoRef* Kit (Bioline). Each 10μL qPCR included 1μM of each primer, 1X SensiMix^TM^*NoRef* reagent, 0.5X SYBR® Green I solution (Bioline), and 1μL template DNA. The reaction conditions were 95°C for 10 min followed by 35 cycles of 95°C for 15 s, 66°C for 30 s and 72°C for 15 s; then a final extension at 72°C for 1 min. This was followed by 50°C for 2 min before melt-curve analysis from 80°C-90°C in 1°C steps. Agarose gel electrophoresis was done to confirm amplification of the expected 265-bp product.

**Table 1:** **Primers and probes usedin qPCR assays**

**Assay**	**Gene**	**Primer and Probe sequences**	**Position**	**Amplicon size (bp)**	**Reference**
Ao	16S rRNA	Forward primer 5'- CTACGCATTTCACCGCTACAC -3'	437-457	265	[33]
		Reverse primer 5'- GGGGAAGAACACGGATAGGA -3'	702-483		
TBL	16S rRNA	Forward primer 5'- TCCTACGGGAGGCAGCAGT -3'	331-349	466	[6,36]
		Reverse primer 5'- GGACTACCAGGGTATCTAATCCTGTT -3'	797-772		
Hi	*hpd*	Forward primer 5’- GGTTAAATATGCCGATGGTGTTG -3’	822-844	151	[37,38]
		Reverse primer 5’- TGCATCTTTACGCACGGTGTA -3’	972-953		
		Probe 5’Hex- TTGTGTACACTCCGT “T” GGTAAAAGAACTTGCAC -SpC6 -3’*	928-896		
Spn	*lytA*	Forward primer 5'- TCTTACGCAATCTAGCAGATGAAGC -3'	306-326	101	[6]
		Reverse primer 5'- GTTGTTTGGTTGGTTATTCGTGC -3'	406-386		
		Probe 5'- [6-FAM]-TTTGCCGAAAACGCTTGATACAGGG -[TAMRA] -3'	354-330		
Mc	*copB*	Forward primer 5'- GTGAGTGCCGCTTTTACAACC -3'	50-70	72	[6]
		Reverse primer 5'- TGTATCGCCTGCCAAGACAA -3'	121-102		

Ao = *A. otitidis*. TBL = Total bacterial load. Spn = *S. pneumoniae*. Hi = *H. influenzae*. Mc = *M. catarrhalis*. 16S rRNA gene position numbers are based on *E. coli* numbering. * Inverted commas in the primer sequence of the *hpd* probe indicate the position of the Black Hole Quencher.

The standard curve (100pg-100fg) was prepared using genomic DNA from the *A. otitidis* reference isolate ATCC51267. The limit of detection was 100fg of genomic DNA, which is equivalent to ~50 genome copies based on an *A. otitidis* genome size of ~1.7-Mb [34]. The *A. otitidis* qPCR efficiency ranged from 0.87-0.90 and all standard curves had R^2^ values >0.99. The no template control was negative in all except two instances in which non-specific amplification was detected below the assay’s limit of detection – quantification cycle (Cq) 32-33 which was at least 5 cycles beyond the limit of detection, at a concentration less than one cell, suggesting detection of primer-dimers.

For swabs, positive results were defined as Cq-value less than or equal to that of the 100fg standard with a dissociation temperature (as determined by melt-curve analysis) within 0.5°C of the mean dissociation temperature of the assay’s standards. Negative results were recorded for samples with i) no amplification; or ii) amplification but dissociation temperature >0.5°C different to the mean dissociation temperature of the standards, consistent with primer-dimer or other non-specific amplicons. Results were considered equivocal when i) replicates gave a positive and a negative result; or ii) Cq-values were greater than that of the 100fg control but melt profiles were consistent with *A. otitidis*, suggesting detection of <50 cells. Equivocal PCRs were repeated with 2μL template DNA. Samples that remained equivocal upon repeat testing were recorded as *A. otitidis* negative.

### Other bacterial load qPCR assays

*H. influenzae*, *S. pneumoniae*, *M. catarrhalis* and total bacterial loads were estimated as previously described [6,35,38] with modification as described in Additional file 1. Primers and probes for all assays are shown in Table 1.

### *A. otitidis* culture and identification

Swabs qPCR-positive for *A. otitidis* were thawed on ice and vortexed. 10μL was then inoculated onto horse blood agar (Oxoid), and brain-heart-infusion agar (BHI; Oxoid) supplemented with 6.5% NaCl (Crown Scientific). Horse blood agar was selected as it had previously been used to culture *A. otitidis* from middle ear specimens [8]. As *A. otitidis* is salt tolerant [7], BHI agar with 6.5% NaCl was included as a potentially selective medium, as previously proposed by Tano *et al.*[9].

*A. otitidis* reference strain ATCC51267 was used as a positive control. Plates were incubated at 37°C in an ambient atmosphere and were read after 2, 5, 7, 9, 14 and 21 days of incubation. Small, off-white colonies [8] of similar appearance to the *A. otitidis* reference isolate were Gram stained and subcultured on horse blood agar with incubation at 37°C for 48 hrs in an ambient atmosphere. Alpha-haemolytic, catalase-positive, oxidase-negative, Gram-positive cocci were considered presumptive *A. otitidis*[7]. Genomic DNA from presumptive *A. otitidis* colonies was extracted and identification was confirmed using the qPCR described above.

### *A. otitidis* antibiotic susceptibility testing

Penicillin, erythromycin and azithromycin MICs were determined using E-Tests® as previously described [8]. Briefly, isolates were suspended in sterile saline to no. 3 MacFarland standard then inoculated onto horse blood agar plates. After drying, E-test® strips were applied and the plates incubated at 37°C in an ambient atmosphere for 48 hrs. These conditions are required to support sufficient *A. otitidis* growth to enable MIC determination [8]. As CLSI MIC breakpoints have not been determined for *A. otitidis,* the breakpoints of *S. pneumoniae*[28] were applied (consistent with previous studies [8,29]).

### Culture for other bacterial species

Culture data for other bacterial species in *A. otitidis* qPCR-positive swabs were derived from the original RCT database. Culture had been performed as previously described [5,30] for 18-24 hrs and did not include testing for *A. otitidis*.

## Results

### qPCR detection of *A. otitidis*

*A. otitidis* was detected by qPCR in ear discharge swabs from 10 of 27 children (37%), including one child with bilateral detection (11 positive ear discharge swabs in total). In the remaining three children with bilateral perforation, two were qPCR-positive for *A. otitidis* in one ear only; and one was *A. otitidis* negative. As part of the original RCT, five of the *A. otitidis*-positive children were treated with azithromycin and five treated with amoxycillin. When reviewed 6-11 days after treatment, 7 of the 10 *A. otitidis*-positive children continued to have a diagnosis of acute otitis media with perforation (3 azithromycin treated, 4 amoxycillin treated). *A. otitidis* was not detected by qPCR in nasopharyngeal swabs (0/27). No further testing of nasopharyngeal swabs was performed.

### Culture of *A. otitidis* qPCR-positive swabs

*A. otitidis* was isolated from 5/11 qPCR-positive ear discharge swabs from 4 children after 2-14 days’ incubation (Table 2). As *A. otitidis* is salt tolerant [7], BHI agar with 6.5% NaCl was used as a selective medium [9]; however, all cultures grew other bacterial species after two days’ incubation, resulting in substantial overgrowth before day 21.

**Table 2:** ***A. otitidis *** **isolates cultured from eardischarge swabs**

**Sample**	**Days of culture until *A. otitidis*** **was isolated**		**Penicillin MIC**	**Erythromycin MIC**	**Azithromycin MIC**
	**HBA agar**	**BHI agar with 6.5%NaCl**			
Child A (Left ear)	5	7	0.19	4	1.5
Child A (Right ear)	2	5	0.125	3	2
Child B	14	5	0.006	>256	128
Child C	Not detected*	9	0.19	>256	>256
Child D	7	Not detected	0.032	96	64

Culture and antibiotic susceptibility data for five isolates from four children (designated child A-D). All cultures were positive for other bacteria after two days’ incubation. MIC values are in μg/mL. In the absence of *A. otitidis*-specific CLSI breakpoints, the MIC values used to define pneumococcal susceptibility were applied [28]. For oral penicillin V: sensitive ≤0.06μg/mL, intermediate susceptibility 0.12-1μg/mL, resistant ≥2μg/mL. For erythromycin: sensitive ≤0.25μg/mL, intermediate susceptibility 0.5μg/mL, resistant ≥1μg/mL. For azithromycin: sensitive ≤0.5μg/mL, intermediate susceptibility 1μg/mL, resistant ≥2μg/mL. HBA = Horse blood agar. *This plate was overgrown by a *Proteus* sp.

### Susceptibility of *A. otitidis* isolates

Five *A. otitidis* isolates were available for antibiotic susceptibility testing (Table 2). Erythromycin MICs ranged from 3 - >256μg/mL. Azithromycin MICs ranged from 1.5 - >256μg/mL. Penicillin MICs ranged from 0.006-0.19μg/mL. If *S. pneumoniae* Clinical Laboratory Standards Institute (CLSI) MIC breakpoints [28] are applied, as previously described [8,29], all isolates were resistant to erythromycin and 4/5 were resistant to azithromycin. Using *S. pneumoniae* CLSI breakpoints for oral penicillin V, 3/5 isolates had intermediate penicillin susceptibility.

### Other bacteria cultured from *A. otitidis*-positive ear discharge swabs

Previously recorded culture data for the 11 *A. otitidis* qPCR-positive swabs were reviewed to determine if other species were present. All *A. otitidis*-qPCR positive swabs were polymicrobial with at least 2-5 other species cultured (Table 3). In one child (Child G), *A. otitidis*, *S. pneumoniae*, *H. influenzae*, *M. catarrhalis*, *Staphylococcus* sp. and other unidentified bacteria were all detected. Overall, *Staphylococcus* sp. were cultured from 10 of the 11 *A. otitidis* qPCR-positive swabs.

**Table 3:** **Other bacteria previously cultured from *A. otitidis *** **qPCR-positive ear discharge specimens**

**Swab**	***S. pneumoniae***	***H. influenzae***	***M. catarrhalis***	**β-haemolytic streptococci**	***Staphylococcus*** **sp.**	***P. aeruginosa***	***Proteus*** **sp***.*	**Other**
Child A (Left ear)	-	+	-	-	-	-	-	+
Child A (Right ear)	-	+	-	-	+	-	-	-
Child B	-	-	n/a	-	+	n/a	+	+
Child C	-	-	n/a	-	+	-	+	+
Child D	-	-	-	+	+	-	-	+
Child E	+	+	-	-	+	-	-	-
Child F	-	-	n/a	-	+	n/a	+	+
Child G	+	+	+	-	+	-	-	+
Child H	-	-	-	-	+	-	-	+
Child I	-	-	-	-	+	+	-	+
Child J	-	-	-	-	+	+	-	+

These data were derived from the original RCT database. Culture data for *M. catarrhalis* and *P. aeruginosa* were not available for some swabs due to overgrowth by *Proteus* species (n/a = not available).

### *A. otitidis* bacterial load and relative abundance in polymicrobial ear discharge specimens

**Figure 1:**
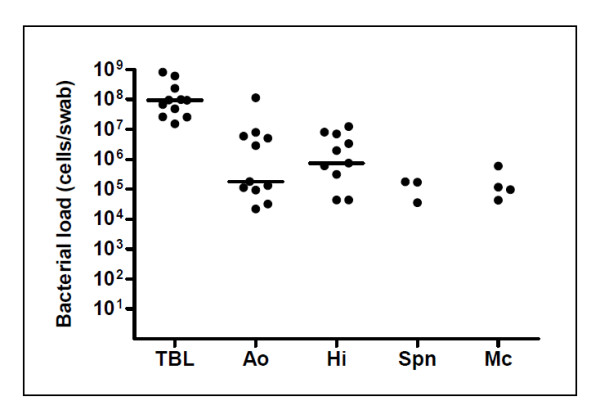
**Total and species-specific bacterial loads in 11 *A. otitidis *-positive ear discharge swabs.** Species-specific data points are only shown for qPCR-positive specimens. *A. otitidis*, *H. influenzae* and total bacterial load median values are indicated with a bar. TBL = Total bacterial load. Ao = *A. otitidis*. Hi = *H. influenzae*. Spn = *S. pneumoniae*. Mc = *M. catarrhalis.*

As all *A. otitidis*-qPCR positive swabs were culture-positive for at least two other species, bacterial load measures were used to determine if *A. otitidis* was a minor or dominant constituent of the bacterial communities (Table 4). *A. otitidis* bacterial load in the 11 qPCR-positive swabs ranged from 2.2 × 10^4^-1.1 × 10^8^ cells/swab (median 1.8 × 10^5^ cells/swab), while the total bacterial load estimates ranged from 1.5 × 10^7^-8.1 × 10^8^ cells/swab (median 9.4 × 10^7^ cells/swab; Figure 1).

**Table 4:** ***H. influenzae *,** ***S. pneumoniae*, and** ***M. catarrhalis*** **bacterial loads in** ***A. otitidis*-positive ear discharge swabs**

	***A. otitidis***	***H. influenzae***	***S. pneumoniae***	***M. catarrhalis***
In *A. otitidis*-positive ED swabs (n = 11^#^)				
qPCR-positive swabs	11	10	3	4
Culture-positive swabs	5	4	2	1
Bacterial load range (cells/swab)	2.2 × 10^4^-1.1 ×10^8^	4.3 ×10^4^-1.2 ×10^7^	3.5 ×10^4^-1.8 ×10^5^	4.3 ×10^4^-5.9 ×10^5^
Number of swabs with bacterial load >1x10^6^ cells/swab	5	5	0	0
Number of swabs culture-positive and bacterial load >1x10^6^ cells/swab	4	3	0	0
Number of swabs with relative abundance <1%	6	5	3	4
Relative abundance range (%)	0.01-0.70	0.02-0.79	0.01-0.68	0.01-0.89
Number of swabs with relative abundance >1%	5	5	0	0
Relative abundance range (%)	2-34	4-27	0	0

^#^One child with bilateral. *A. otitidis culture was only performed for qPCR-positive swabs.

*A. otitidis* relative abundance was then determined by expressing its bacterial load as a percentage of the total bacterial load (Table 4). The relative abundance of *A. otitidis* ranged from 0.01-34% of the total bacterial load (median 0.7%). In 6 of 11 qPCR-positive swabs the *A. otitidis* relative abundance was <1% (median 0.2%). The *A. otitidis* bacterial load in these swabs ranged from 2.2 × 10^4^-1.8 × 10^5^ cells/swab and only one swab was positive by culture. In the remaining five swabs the relative abundance ranged from 2-34% of the total bacterial load (median 9%). These samples had the highest *A. otitidis* bacterial loads (2.9 × 10^6^-1.1 × 10^8^ cells/swab) and 4 of 5 were *A. otitidis*-positive by culture.

### Bacterial load and relative abundance of *A. otitidis* compared to recognised otopathogens

*H. influenzae, S. pneumoniae* and *M. catarrhalis* bacterial loads were determined for the 11 *A. otitidis*-positive ear discharge swabs (Table 4 and Figure 1). Ten *A. otitidis*-positive swabs were also qPCR-positive for *H. influenzae*. Overall, *H. influenzae* and *A. otitidis* bacterial loads and relative abundances were similar (Table 4): the bacterial load ranged from 4.3 × 10^4^-1.2 × 10^7^ cells/swab for *H. influenzae* (median 7.4 × 10^5^ cells/swab) and 2.2 × 10^4^-1.1 × 10^8^ cells/swab for *A. otitidis* (median 1.8 × 10^5^ cells/swab). Maximum relative abundance was 27% for *H. influenzae* versus 34% for *A. otitidis*. The relative abundance of *H. influenzae* was <1% in 5/10 *H. influenzae* qPCR-positive swabs. In 5/10 *H. influenzae* qPCR-positive swabs the *A. otitidis* bacterial load was higher than that of *H. influenzae*.

*S. pneumoniae* and *M. catarrhalis* were detected by qPCR in fewer *A. otitidis*-positive swabs (3/11 and 4/11, respectively) and were only present at <1% relative abundance (Table 4). *S. pneumoniae* bacterial load ranged from 3.5 × 10^4^-1.8 × 10^5^ cells/swab. *M. catarrhalis* bacterial load ranged from 4.3 × 10^4^-5.9 × 10^5^ cells/swab.

## Discussion

### *A. otitidis* is present in the ear discharge of Indigenous children with AOM with perforation

In this study, *A. otitidis* was detected by qPCR in ear discharge swabs from 10 of 27 children (37%). This rate is consistent with PCR-based AOM studies from Finland, the USA and Japan (25%, 32% and 50%, respectively) [13,15,16], and a culture-based study of chronic OME in Indigenous and non-Indigenous Australian children (45% and 36%, respectively) [8] which tested MEF from children referred for myringotomy.

### *A. otitidis* isolation from ear discharge swabs

Published incubation times for *A. otitidis* culture vary from 5-14 days [9,22]. Data from this study support culture for 14 days. It is important to note that unless extended incubation times are used, culture-based otitis media studies may not detect *A. otitidis*.

Using an extended culture procedure, we isolated *A. otitidis* from 5 of 11 qPCR-positive ear discharge swabs. Several factors may have contributed to the low *A. otitidis* isolation rate. Firstly, PCR-positive but culture-negative results may indicate detection of non-viable bacteria; however, it is generally accepted that DNA is quickly cleared from the middle ear [39-41] suggesting that a positive PCR is indicative of viable cells. Secondly, *A. otitidis* viability may have been affected by storage at -70°C for up to five years; however, viability of *S. pneumoniae* and *H. influenzae* has been demonstrated in swabs stored for up to 12 years at -70°C [42] suggesting good overall bacterial survival despite the prolonged storage.

Overgrowth by other bacterial species likely affected *A. otitidis* isolation. All cultures grew other species after two days’ incubation, which potentially obscured the small *A. otitidis* colonies. Use of BHI agar with 6.5% NaCl as a selective medium was unsuccessful due to heavy overgrowth by other species after two days’ incubation, despite the high salt concentration. Interestingly, successful *A. otitidis* culture was associated with higher bacterial loads (4 of 5 culture-positive swabs with bacterial load ≥1 × 10^6^cells/swab versus 1/6 culture-positive with bacterial load <1 × 10^6^cells/swab). The small study size limits further interpretation of this finding. Overall, our findings are consistent with the lower sensitivity of culture compared to PCR that has been documented for several bacteria in middle ear samples [43] and highlights the difficulties associated with *A. otitidis* culture, even when prolonged culture conditions are used.

### Macrolide resistance in *A. otitidis* isolates

The five *A. otitidis* isolates had MIC values suggestive of macrolide resistance (erythromycin MICs 3 to >256μg/mL; azithromycin MICs 1.5 to >256μg/mL). If *S. pneumoniae* CLSI breakpoints are applied [8,29], 3 of 5 *A. otitidis* isolates also had intermediate level penicillin resistance. The validity of applying *S. pneumoniae* breakpoints to interpret *A. otitidis* MIC data remains to be determined, especially as atypical culture conditions are required to achieve sufficient growth [8]. Specific CLSI breakpoints are required to confirm macrolide resistance and reduced-penicillin susceptibility in *A. otitidis* isolates. Overall, our findings are consistent with previous studies describing macrolide resistance in *A. otitidis*[8,29].

### Is *A. otitidis* contributing to middle ear pathology?

The clinical significance of *A. otitidis* in the ear discharge swabs is unclear. Several studies have found *A. otitidis* in the ear canal of healthy volunteers indicating that it is part of the normal canal flora [9,24-26]. Other studies using culture or species-specific PCRs have detected *A. otitidis* (in the absence of recognised pathogens) in MEF from children with otitis media collected after careful disinfection of the canal and tympanic membrane suggesting infection of the middle ear [8,27].

In this study, *A. otitidis* was not detected by PCR in any nasopharyngeal swabs. Previous PCR and culture-based studies from other populations have reported *A. otitidis* in 7-12% of nasopharyngeal swabs [9,16,23]. As our study was limited to 27 children, it is possible that *A. otitidis* nasopharyngeal carriage may be detected if a larger cohort were tested. However, failure to detect *A. otitidis* in nasopharyngeal swabs from any of the children with positive ear discharge swabs suggests it is unlikely to be a primary otopathogen in this population.

This prompts further consideration of *A. otitidis* in the canal flora. Staphylococcal sp. were cultured from 10 of the 11 *A. otitidis* qPCR-positive ear discharge swabs. This may indicate detection of canal flora; however, it was not possible to confirm this as paired canal swabs were not available. Furthermore, as the study children may have had a perforation for up to 6 weeks, secondary middle ear infection by these species cannot be excluded.

Bacterial load and relative abundance measures were used to better understand the significance of *A. otitidis* in the polymicrobial ear discharge swabs. *A. otitidis* bacterial load and relative abundance measures were similar to that of the otopathogen *H. influenzae* (in *A. otitidis*-positive swabs). For both species, relative abundance <1% was associated with bacterial load <10^6^ cells/swab and most of these samples were qPCR-positive and culture-negative. This suggests a minor role for these species in these specimens, and may be indicative of canal flora (*H. influenzae* has also been reported in normal canal flora from healthy volunteers [26]). For the remaining specimens, relative abundance ranged from 2-34% and 4-27% for *A. otitidis* and *H. influenzae*, respectively. The significance of this finding is unclear as there are no data describing relative abundance thresholds, and the small size of this study limits further interpretation. However, a relative abundance of 34% is clearly suggestive of a dominant species. Furthermore, *A. otitidis* was present at a relative abundance greater than that of *H. influenzae* in 5/10 swabs positive for both species. These data suggest that, in these children, *A. otitidis* may have secondarily infected the middle ear following tympanic membrane perforation. This interpretation is consistent with the hypothesis previously proposed by De Baere *et al.*[26] who reported substantially higher rates of *A. otitidis* in ear canal rather than nasopharyngeal swabs (83% versus 8%) and concluded that *A. otitidis* in MEF from children with OME most likely reflected either secondary infection following perforation or specimen contamination by canal flora. As Indigenous Australian children are at high-risk of progressing to CSOM [44], further research is warranted to better understand *A. otitidis* in secondary middle ear infection, particularly where macrolide antibiotics, such as azithromycin, are used to treat AOM with perforation [5].

### Study limitations

This study retrospectively considered *A. otitidis* in polymicrobial ear discharge swabs collected from Indigenous Australian children with AOM with perforation. The study specimens had been stored for up to five years. While it is unclear if prolonged storage may have adversely affected qPCR detection of *A. otitidis*, culture-based studies have reported continued bacterial viability in upper respiratory swabs after up to 12 years storage at -70°C [42].

The study focused on Indigenous Australian children who are at high-risk of progression to CSOM, and thus the findings may not be generalisable to other populations. The small study size (27 children) also limits generalisation of the findings. Despite these limitations, it appears that *A. otitidis* is often present in the ear discharge of young Indigenous children with AOM with perforation (37% of our cohort).

The study was also potentially limited by the ear discharge sampling technique. Ear discharge swabs in this study were all collected from children with AOM with perforation affecting <2% of the pars tensa of the tympanic membrane for up to six weeks. Paired canal swabs were not collected. Canals were cleaned prior to sampling, but disinfection was not done. As the small size of the perforations prevented middle ear sampling, it is likely that some degree of contamination from canal flora will be present in the swabs. This limitation prevents definitive differentiation of canal flora from middle ear pathogens.

The qPCR described in this study is a modification of a previously published qualitative *A. otitidis* PCR [33]. The qPCR had efficiency of 0.87-0.90, slightly below the ideal range of 0.9-1.10 [45]. This may reflect the amplicon size which is approximately twice that recommended for qPCR [45]. This limitation was accepted as there are few data describing alternative *A. otitidis*-specific PCR targets and a qPCR efficiency of 0.8-0.9 is common to other bacterial load assays [46].

## Conclusions

This study used qPCR to show that *A. otitidis* is present in ear discharge swabs from Indigenous Australian children with AOM with perforation. In a subset of ear discharge swabs we found high *A. otitidis* load and relative abundance. The absence of *A. otitidis* in nasopharyngeal swabs suggests *A. otitidis* may only have a role in secondary middle ear infection following tympanic membrane perforation. Larger longitudinal studies and treatment trials should include paired ear discharge, canal and nasopharyngeal swabs to further test this hypothesis. Such studies should be PCR-based and include culture to assess antibiotic susceptibility. Bacterial load and relative abundance estimates should be considered when investigating the significance of *A. otitidis* in polymicrobial contexts, particularly in children with existing perforations. In light of the co-detection of *Staphylococcus* sp. in 10 of the 11 *A. otitidis* qPCR-positive specimens, future studies should also consider the potential for polymicrobial secondary middle ear infection by these and other species. Where collection of MEF by tympanocentesis is not feasible, paired ear discharge and canal swabs may be helpful in distinguishing bacteria from the outer and middle ear. Additional studies investigating a role for *A. otitidis* as a secondary pathogen in CSOM are also warranted in populations with a high prevalence of spontaneous tympanic membrane perforation.

## Abbreviations

AOM: Acute otitis media; BHI: Brain heart infusion agar; CLSI: Clinical Laboratory Standards Institute; Cq: Quantification cycle; CSOM: Chronic suppurative otitis media; RCT: Randomised controlled trial; MEF: Middle ear fluid; MIC: Minimum inhibitory concentration; OME: Otitis media with effusion; qPCR: Quantitative PCR.

## Competing interests

The authors declare that they have no competing interests.

## Authors' contributions

RLM conceived the study, coordinated laboratory testing and prepared the manuscript. MJB participated in the study design, qPCR development, data analyses and manuscript preparation. JB and PC carried out the qPCR and assisted with data analyses. PSM and AJL participated in the study design and provided critical review of the manuscript. HSV participated in the study design, co-ordination and manuscript preparation. All authors read and approved the final manuscript.

## Pre-publication history

The pre-publication history for this paper can be accessed here:

http://www.biomedcentral.com/1472-6815/12/11/prepub

## Supplementary Material

Additional file 1 **Other bacterial load qPCR assays.** qPCR methods used to quantify *H. influenzae*, *S. pneumoniae*, *M. catarrhalis* and total bacterial loads. Click here for file
