# Supplementary material for: Quantitative PCR of ear discharge from Indigenous Australian children with acute otitis media with perforation supports a role for Alloiococcus otitidis as a secondary pathogen
Source: BMC Ear Nose Throat Disord. 2012 Oct 3;12:11. doi: 10.1186/1472-6815-12-11 (PMC3546424; doi:10.1186/1472-6815-12-11)
Supplement: Additional file 1 — Other bacterial load qPCR assays. qPCR methods used to quantify H. influenzae, S. pneumoniae, M. catarrhalis and total bacterial loads. [file 1472-6815-12-11-S1.doc]

**Total bacterial load qPCR**

Total bacterial load was estimated using the primers of Nadkarni *et al*[1] which amplify a 466-bp region between positions 331-797 of the 16S rRNA gene, based on *E. coli* numbering. The assay was performed as previously described[2,3], with the following modifications. The assay was done with SYBR® Green instead of a probe[1] to improve the qPCR efficiency. Each 10µL qPCR reaction included 1X SensiMix^TM^ SYBR® reagent (Bioline), 300nM of each primer and 1µL of template DNA. The reaction conditions were an initial hold at 50ºC for 2 min followed by incubation at 95ºC for 10 min then 35 cycles of 95ºC for 15 s, 58ºC for 15 s and 72ºC for 45 s. Melt-curve analysis was then done between 80ºC-90ºC with 0.1ºC steps. As multiple amplicons form during universal 16S rRNA gene amplification, it was not possible to define a single melt-curve dissociation temperature which could be used to differentiate specific from non-specific amplicons. To overcome this limitation, the entire melt-curve was considered. Replicate analyses with irreproducible melt-curves were considered indicative of non-specific amplification. As expected[1], some amplification was detected in the total bacterial load qPCR no template control (Cq 33-36). This amplification was detected >5 cycles beyond the limit of detection, with concentration equivalent to <10 cells.

Genomic DNA from the *S. pneumoniae* ATCC49619 reference isolate was used to prepare the standard curve (2000ng-200fg). Thus, the assay estimated total bacterial load assuming four ribosomal operons per cell[1,3]. The total bacterial load qPCR limit of detection was 90 cells based on an *S. pneumoniae* genome size of ~2Mb [GenBank:AE005672]. The total bacterial load qPCR efficiency was 0.81 and the R^2^ value was 0.996.

## *H. influenzae* qPCR

*H. influenzae* qPCR used the probe and primers described by Wang *et al*[4] which amplify a 151-bp region between positions 822-972 of the *hpd* gene [GenBank:GQ201998] with the hydrolysis probe located at position 896-928. Each 10µL qPCR reaction mix included 1X Taqman® Universal Master Mix (Applied Biosystems), 100nM forward primer, 200nM reverse primer, 100nM probe, and 1µL of template DNA. The reaction conditions were an initial hold at 50ºC for 2 min followed by incubation at 95ºC for 10 min then 40 cycles of 95ºC for 15 s and 60ºC for 60 s. Genomic DNA from the *H. influenzae* reference isolate ATCC19418 was used to prepare the standard curve (2000ng-200fg). The limit of detection was 10 cells based on *H. influenzae* genome size of ~1.8-Mb [GenBank: [CP000671](http://www.ncbi.nlm.nih.gov/nuccore/CP000671)]. The *H. influenzae* qPCR efficiency was 0.86 and the R^2^ value was 0.999. Amplification was not detected in the no template control.

## *S. pneumoniae* qPCR

The *S. pneumoniae* qPCR was performed using the probe and primers described by Smith-Vaughan *et al*[2] which amplify a 101-bp region between positions 306-406 of the *lytA* gene [GenBank: AY204888.1] with the hydrolysis probe located between position 330-354. The assay was performed as previously described[2,3] with the following modifications. Each 10µL qPCR reaction mix included 1X Taqman® Universal Master Mix (Applied Biosystems), 100nM of each primer, 200nM of probe, and 1µL of template DNA. The reaction conditions were an initial hold at 50ºC for 2 min followed by incubation at 95ºC for 10 min, then 40 cycles of 95ºC for 15 s, 58ºC for 25 s and 72ºC for 35 s. Genomic DNA from the *S. pneumoniae* reference isolate ATCC49619 was used to prepare the standard curve (2000ng-200fg). The limit of detection was 9 cells based on *S. pneumoniae* genome size of ~2Mb [GenBank: AE005672]. The *S. pneumoniae* qPCR efficiency was 0.80 and the R^2^ value was 0.997. Amplification was not detected in the no template control.

## *Moraxella catarrhalis* qPCR

The *M. catarrhalis* qPCR was performed using primers previously described by Smith-Vaughan *et al*[2] which amplify a 72-bp region between positions 50-121 of the *copB* gene [GenBank: U69982]. The assay was performed as previously described[2,3], with the following modifications. The assay was done with SYBR® Green instead of a probe to improve the qPCR efficiency. Each 10µL qPCR reaction mix included 1X SensiMix^TM^ SYBR® reagent, 300nM of each primer and 1µL of template DNA. The reaction conditions were an initial hold at 50ºC for 2 min followed by incubation at 95ºC for 10 min, then 40 cycles of 95ºC for 15 s, 58ºC for 25 s and 72ºC for 35 s, followed by a melt-curve analysis between 75ºC-95ºC with 0.1ºC steps. Melt-curve analysis was interpreted as described for the *A. otitidis* qPCR (above). Genomic DNA from the *M. catarrhalis* reference isolate ATCC8176 was used to prepare the standard curve (2000ng-200fg). The assay’s limit of detection was 10 cells based on *M. catarrhalis* genome size of ~1.8Mb [GenBank: CP002005]. The *M. catarrhalis* qPCR efficiency was 0.93 and the R^2^ value was 0.999. Amplification was not detected in the no template control.

**References**

1. Nadkarni MA, Martin FE, Jacques NA, Hunter N: Determination of bacterial load by real-time PCR using a broad-range (universal) probe and primers set. *Microbiology* 2002, 148: 257-266.

2. Smith-Vaughan H, Byun R, Nadkarni M, Jacques NA, Hunter N, Halpin S, Morris PS, Leach AJ.: Measuring nasal bacterial load and its association with otitis media. *BMC Ear Nose and Throat Disord* 2006, 10: 10.

3. Binks M, Cheng A, Smith-Vaughan H, Sloots T, Nissen M, Whiley D, McDonnell J, Leach A.: Viral-bacterial co-infection in Australian Indigenous children with acute otitis media. *BMC Infectious Diseases* 2011, 11: 161.

4. Wang X, Mair R, Hatcher C, Theodore MJ, Edmond K, Wu HM, Harcourt BH, Carvalho M, Pimenta F, Nymadawa P, Altantsetseg D, Kirsch M, Satola SW, Cohn A, Messonnier NE, Mayer LW.: Detection of bacterial pathogens in Mongolia meningitis surveillance with a new real-time PCR assay to detect *Haemophilus influenzae*. *Int J Med Microbiol* 2011, 301: 303-309.
